# High dose brachytherapy with non sealed ^188^Re (rhenium) resin in patients with non-melanoma skin cancers (NMSCs): single center preliminary results.

**DOI:** 10.1007/s00259-020-05088-z

**Published:** 2020-11-02

**Authors:** Paolo Castellucci, F. Savoia, A. Farina, G. M. Lima, A. Patrizi, C. Baraldi, F. Zagni, S. Vichi, C. Pettinato, A. G. Morganti, L. Strigari, S. Fanti

**Affiliations:** 1 Nuclear Medicine, IRCCS Azienda Ospedaliero-Universitaria di Bologna, Via Massarenti 9, 40138 Bologna, Italy; 2 Dermatology, IRCCS Azienda Ospedaliero-Universitaria di Bologna, Via Massarenti 9, 40138 Bologna, Italy; 3 Medical Physics, IRCCS Azienda Ospedaliero-Universitaria di Bologna, Via Massarenti 9, 40138 Bologna, Italy; 4 Department of Industrial Engineering, Azienda Ospedaliero-Universitaria di Bologna, Via Massarenti 9, 40138 Bologna, Italy; 5 Medical Physics Unit, Fondazione IRCCS Ca Granda Ospedale Maggiore Milano, Milan, Italy; 6 Radiation Oncology, IRCCS Azienda Ospedaliero-Universitaria di Bologna, Via Massarenti 9, 40138 Bologna, Italy

**Keywords:** Non-melanoma skin cancers, Brachytherapy, ^188^Rhenium, Not- sealed sources

## Abstract

**Background and aim:**

High dose brachytherapy using a non sealed ^188^Re-resin (Rhenium-SCT®, Oncobeta® GmbH, Munich, Germany) is a treatment option for non-melanoma skin cancer (NMSC). The aim of this prospective study was to assess the efficacy and the safety of a single application of Rhenium-SCT® in NMSC.

**Materials and method:**

Fifty consecutive patients (15F, 35 M, range of age 56–97, mean 81) showing 60 histologically proven NMSCs were enrolled and treated with the Rhenium-SCT® between October 2017 and January 2020. Lesions were located on the face, ears, nose or scalp (*n* = 46), extremities (*n* = 9), and trunk (*n* = 5). Mean surface areas were 7.0 cm^2^ (1–36 cm^2^), mean thickness invasion was 1.1 mm (0.2–2.5 mm), and mean treatment time was 79 min (21–85 min). Superficial, mean, and target absorbed dose were 185 Gy, 63 Gy, and 31 Gy respectively. Patients were followed-up at 14, 30, 60, 90, and 180 days posttreatment, when dermoscopy and biopsy were performed. Mean follow-up was 20 months (range 3–33 months). Early skin toxicity was classified according to Common Terminology Criteria for Adverse Events (CTCAE). Cosmetic results were evaluated after at least 12 months according to Radiation Therapy Oncology Group (RTOG) scale.

**Results:**

At 6 months follow-up, histology and dermoscopy were available for 54/60 lesions, of which 53/54 (98%) completely responded. One patient showed a 1-cm^2^ residual lesion that was subsequently surgically excised. Twelve months after treatment, 41/41 evaluable lesions were free from relapse. Twenty four months after treatment, 23/24 evaluable lesions were free of relapse. In 56/60 lesions early side effects, resolving within 32 days were classified as grades 1–2 (CTCAE). In the remaining 4/60 lesions, these findings were classified as grade 3 (CTCAE) and lasted up to 8–12 weeks but all resolved within 90 days. After at least 12 months (12–33 months), cosmetic results were excellent (30 lesions) or good (11 lesions).

**Conclusion:**

High dose brachytherapy with Rhenium-SCT® is a noninvasive, reasonably safe, easy to perform, effective and well-tolerated approach to treat NMSCs, and it seems to be a useful alternative option when surgery or radiation therapy are difficult to perform or not recommended. In our population 98% of the treated lesions resolved completely after a single application and only one relapsed after 2 years. Larger patients’ population and longer follow-up are needed to confirm these preliminary data and to find the optimal dose to administer in order to achieve complete response without significant side effects.

## Introduction

Non-melanoma skin cancers (NMSCs) are the most common cancers in humans and represent about 80% of all skin cancer cases, with more than 3 million patients treated every year. [1] Basal cell carcinoma (BCC) is the most frequent NMSC, accounting for 70% of cases, while squamous cell carcinoma (SCC) accounts for 20%, although its incidence is rising.

Risk factors for NMCSs are: fair skin phototype, chronic sun exposure, old age, immunosuppression, and HPV infection.

Most NMSCs are located on areas more exposed to sun light, in particular, the scalp, face, and hand dorsum [1].

The treatment of choice is in most cases surgery. Mohs micrographic surgery (MMS) is currently considered the best option for primary NMSC demonstrating a 5-year cure rate higher than 95% in both BCCs [2] and SCCs [3]. However, the main limitation to MMS is represented by patients with large or multiple lesions localized in areas where radical surgical approach is technically difficult or disfiguring. These may include the nose-wing, ears, eyelids, lips, external genitals, or fingers. In these cases, the results of surgery may be suboptimal in terms of radicality and cosmetic results, while also reducing the functionality of the treated areas [4].

For elderly patients, the choice of therapy also depends on the patient general health condition, mental health, life expectancy, and personal preference; therefore, treatment modalities other than surgery should be carefully considered. Nonsurgical treatment options of NMSCs include cryo-therapy, topical medication such as imiquimod and 5-fluorouracil, photodynamic therapy, curettage and electrodessication, laser-therapy, and electronic brachytherapy [4].

Electronic brachytherapy with sealed solid sources is an alternative method that showed excellent results. Its limitation is mainly due to the difficulty to treat large lesions or lesions with nonplanar concave or convex surfaces (the nose, ears, lips, external genitals) [5].

High dose brachytherapy using a nonsealed rhenium-188 resin, commercially known as Rhenium-SCT® (Oncobeta® GmbH, Munich, Germany), is a new treatment option that makes it possible to bring radioactivity as close as possible to the whole surface of the lesion independently of its three-dimensional shape and size.

This brachytherapy technique is based on the property of ^188^Re to release a high energy, emitting 85% beta and 15% gamma radiation (Beta 2.2 MeV; Gamma 155KeV).^188^rhenium releases 92% of its energy within 2-mm depth in the skin. [6]

Brachytherapy with ^188^Re may have a clinical role as a tailored treatment in cases where (a) surgery or EBRT or other brachytherapy approaches would be suboptimal with regard to the location, the extent of the lesion or the cosmetic outcome that may result from skin surgery; (b) patients would not be eligible for surgery considering their general health condition and comorbidities; and (c) patients who refuse surgery. The limited literature on this subject does not allow for a systematic analysis of the results of this method, which however appears to be very promising and to date has provided excellent results in terms of long-term outcome and absence of significant long-term side effects [7–10].

We present the preliminary results of our first experience (from October 2017 to January 2020) on the use of this technique in a population of patients affected by NMSCs. The main goal was to assess the clinical efficacy and safety of a single application of a standardized high dose brachytherapy using a nonsealed ^188^Re source in the treatment of NMSCs.

## Material and methods

The study was performed according to the Helsinki Declaration, patients signed written informed consent to participate, and the study was approved by local Ethical Committee (23/2019/Oss/AOUBo). Between October 2017 and January 2020, patients affected by NMSC (including both new diagnosis and relapses) were selected by the Dermatology Unit of the Azienda Ospedaliero-Univarsitaria of Bologna, S. Orsola–Malpighi Hospital.

Inclusion criteria of our study were (1) histologically proven cutaneous BCC or SCC; (2) lesion thickness invasion not deeper than 2.5 mm (arbitrary cutoff based on ^188^Re characteristics) according to single or multiple diagnostic biopsies; (3) lesions located in the scalp, face, ears, or fingers or other areas in which surgery, EBRT or standard brachytherapy would have been difficult to perform; and (4) contraindication or refusal of surgery.

### Population characteristics

Between October 2017 and January 2020, 50 consecutive patients (15F, 35 M, range of age 56–93 years; mean 81) showing 60 histologically proven NMSCs (41BCC; 18SCC; 1BCC&SCC) were enrolled. Lesions were located on the face, ears, nose or scalp (46), extremities (9), and trunk (5). Mean surface area was 7.0 cm^2^ (range 1–36 cm^2^) and mean thickness invasion 1.1 mm (range 0.2–2.5 mm). Mean treatment time was 79 min (range 21–285 min). In our population, 18 out of 60 lesions had already been treated with other therapies and relapsed (five lesions had already received surgery; two lesions surgery and photodynamic therapy or cryotherapy; ten lesions had already received cryo-therapy, laser and photodynamic therapy; one imiquimod) while 42 lesions were new diagnoses at presentation.

Patients’ characteristics are reported in Table 1.

**Table 1:** Patient population

Population details			
Patients		Lesions	
Num. of patients	50	Num. of NMSCs lesions	60
Age (years)—mean and range	81 (56–97)	BCC	41 (70%)
M/F	35/15	SCC	18 (25%)
Follow-up (months)—mean and range	18 (3–30)	BCC and SCC	1 (2%)
Localization		Lesions characteristics	
Head (face and scalp)	46 (76%)	Surface area (cm^2^) mean (range)	7.0 (1–36)
Extremities 9 (15%)		Thickness invasion (mm) mean (range)	1.1 (0.2–2.5)
		Volume (cm^3^) mean (range)	0.7 (0.05–7.2)
Trunk	5 (9%)	Previously treated	18 (33%)
^188^ Re Administered	Mean 335 MBq (range 48–1028)	Treatment time	Mean 78 min (range 21–285)

*NMSC* nonmelanoma skin cancer; *BCC* basal cell carcinoma; *SCC* squamous cell carcinoma

### Follow-up

Patients were followed-up after 14–30–60–90–180 days from the treatment and then every 90–180 days up to 33 months.

### Standard of reference

Six months after Rhenium-SCT® treatment, patients were classified as complete responders (CR) if the dermoscopy did not show any suspected area of persistence of the disease that may deserve a biopsy or if the biopsy guided by the dermoscopy resulted negative; partial responders (PR) if the biopsy on a suspected area resulted positive but the treatment with Rhenium-SCT® caused a significant reduction in the extent of the lesion making possible the surgical excision or other local therapies with subsequent complete histological response; nonresponders (NR) in case of disease persistence.

### Skin toxicity and cosmetic results

Two expert clinicians have classified early skin toxicity: a dermatologist (F.S.) and a radiation oncologist (A.G.M.). Early skin toxicity has been evaluated according to Common Terminology Criteria for Adverse Events (CTCAE 5.0) [11] within the first 30 days in all 60 lesions **(**Table 2). Cosmetic results have been evaluated after at least 12 months (range 12–33 months) in 41 evaluable lesions according to Radiation Therapy Oncology Group criteria (RTOG) [12] (Table 3).

**Table 2:** Skin toxicity according to CTCAE 5.0 [11]

Skin Toxicity	G1	G2	G3	G4	G5
Atrophy	Mild	Marked			
Alopecia	< 50%	> 50%			
Pigmentation change	Mild or localized	Marked or generalized			
Erythema	Mild	Moderate	Severe	Necrosis	Death
Skin ulceration	< 1 cm	1–2 cm	> 2 cm	Deep structures involved	Death

**Table 3:** Cosmetic scale according to RTOG [12]

Cosmetic scale	Definition
Excellent	No changes, to slight atrophy or pigment change, or slight hair loss or no changes to slight induration or loss of subcutaneous fat
Good	Patch atrophy, moderate telangiectasia, and total hair loss; moderate fibrosis but asymptomatic; slight field contracture with less than 10% linear reduction
Fair	Marked atrophy and gross telangiectasia; severe induration or loss of subcutaneous tissue, field contracture greater than 10% linear measurement
Poor	Ulceration or necrosis

### Therapy details

The treatment consisted in a ^188^Re-based resin application using a dedicated device (Rhenium-SCT®, Skin Cancer Therapy, Oncobeta® GmbH, Germany) provided with a carpoule filled with radioactive ^188^Re resin. The radioactive resin is applied over a 7-μm foil placed over the skin lesion to avoid any direct contact of the resin with the skin.

**Fig. 1:**
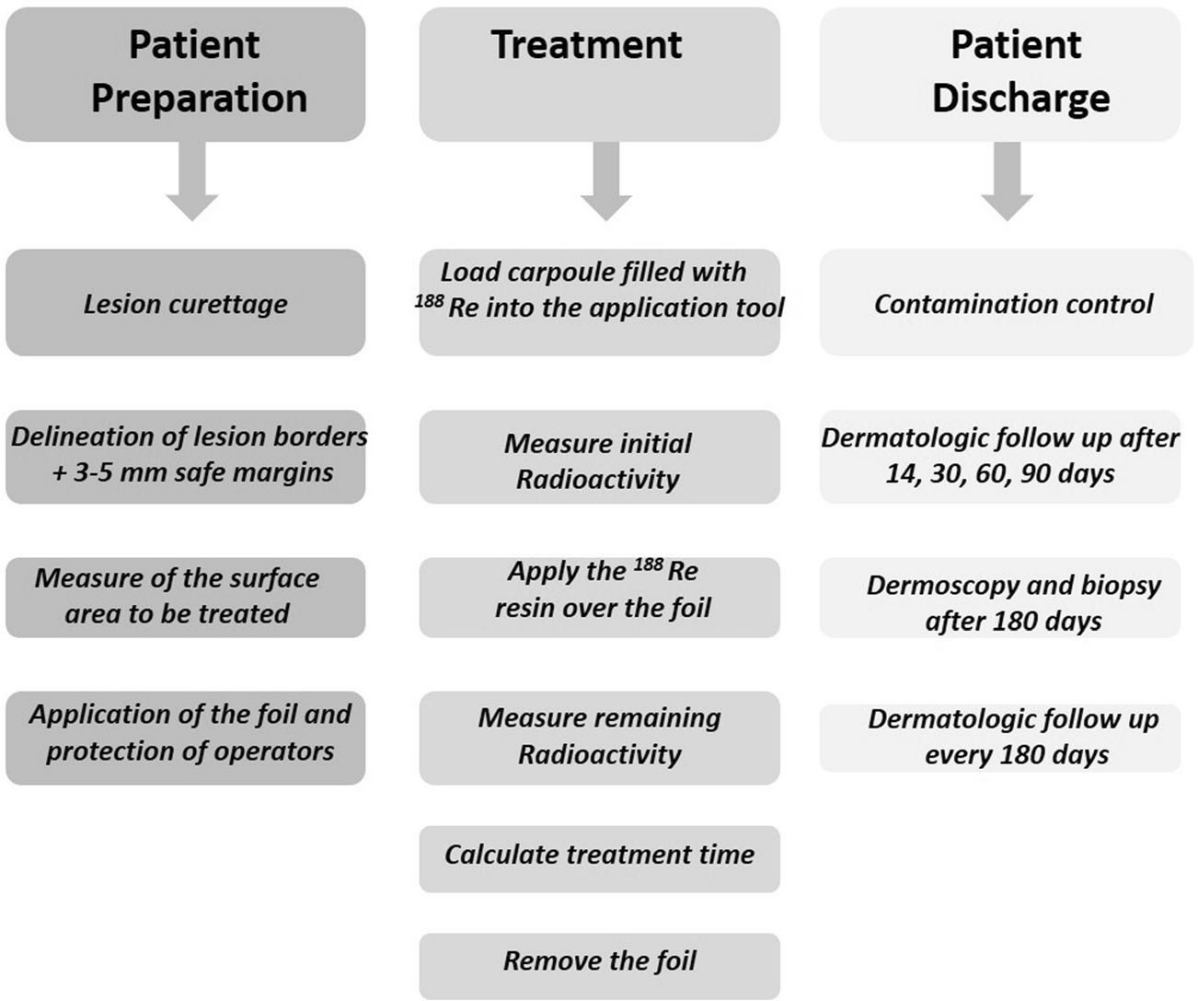
Patient’s preparation, patient’s treatment and patient’s discharge and follow-up

The steps required for patient preparation, administration of therapy, and patient discharge and follow-up are summarized in Fig. 1.

The treatment goal was to deliver an adjusted normalized adsorbed dose to each single lesion to the deepest point of neoplastic infiltration (target dose) assessed by one or multiple pre-treatment biopsies in order to avoid retreatments. Calculation of the estimated dose on a single lesion has been performed using two independent methods: Varskin5 software [13] and the Monte Carlo Code Fluka [14]. Both methods allow assessing the dose distribution taking into account the energy spectrum of ^188^Re, the thickness of the invasion, the surface of the lesion, the activity dispensed, and the duration of the treatment. In all the cases considered, the two methods result to be consistent.

In accordance with the previously reported data [7–10], the first 10 treated lesions were treated with an empiric mean target dose of 47 Gy to the deepest point of neoplastic invasion. Consequently, this resulted in a mean adsorbed dose to the whole volume of the lesion (mean dose) of 92 Gy and in a mean adsorbed dose at 0.01 mm of neoplastic invasion (superficial dose) of 260 Gy. Given the excellent response rate, but the not negligible incidence of early side-effects (see the “Results” paragraph), we proceeded to a progressive reduction of the delivered doses, we established a dose deescalation protocol using the target dose and the mean dose as indicators. Summarizing, the mean values of the adsorbed doses delivered were ten lesions received a target dose of 47 Gy, a mean dose of 92 Gy, and a superficial dose of 260 Gy; twenty-three lesions received a target dose of 35 Gy, a mean dose of 65 Gy, and a superficial dose of 185 Gy (25% reduction); twenty-seven lesions received a target dose of 23 Gy, a mean dose of 48 Gy, and a superficial dose of 155 Gy (50% reduction). See Table 4.

**Table 4:** Lesions characteristics of the three dose de-escalation groups based on the adsorbed Target Dose

Target dose * Mean dose** Superficial dose*** Deescalation	Number of treated lesions	Treated surface area (cm^2^)	Neoplastic thickness invasion (mm)	Volume (cm^3^)
47 Gy (target dose) 92 Gy (mean dose) 260 Gy (superficial dose)	10	5.8	1.1	0,7
35 Gy (target dose) 66 Gy (mean dose) 185 Gy (superficial dose) 25% deescalation	23	5.3	0.9	0,4
23 Gy (target dose) 48 Gy (mean dose) 155 Gy (superficial dose) 50% deescalation	27	9.0	1.2	1,0

*Target dose: adsorbed dose to the deepest point of neoplastic invasion. **Mean dose: adsorbed dose by the whole volume of the lesion. ***Superficial dose: adsorbed dose at 0.01 mm of neoplastic invasion

### Treatment monitoring

According to our study design, patients were treated with Rhenium-SCT® on day 0 and followed by a dermatological examination on days 14, 30, 60, 90, 180, then every 90–180 days. The response to therapy was evaluated after 6 months, through clinical evaluation and dermoscopy examination, using both manual polarized noncontact dermoscopy (DermLite 3 Gen, San Juan Capistrano, California, USA) and digital nonpolarized contact dermoscopy (Foto Finder dermatoscope®, Teachscreen Software, Bad Birnbach, Germany) followed by a biopsy (if clinically needed).

### Statistical analysis

Univariate and multivariate analysis of the predictive factors of CTCAE G3 acute toxicity was performed using the logistic regression model including all the dosimetric (e.g., target dose, mean dose, superficial dose) and lesion related variables (e.g., treated areas) as continuous, variables. The utility of the identified variables as early predictor of toxicity has been assessed using the area under curve (AUC) of ROC curve. When a perfect correlation of predicted versus observed toxicity was found, the AUC was equal to 1 whereas random assignment of outcome led to a ROC/AUC of 0.5 [15]. The data analysis was performed with R version 3.6.3 [16].

## Results

Six months after Rhenium-SCT® treatment, 54 evaluable lesions have been studied with dermoscopy and/or histology after biopsy. In 49/54 lesions, a dermoscopy followed by a biopsy have been performed while in 5/54 dermoscopy did not show any suspicious finding that could guide to a biopsy, therefore the biopsy was not performed. According to these diagnostic tests, 53 out of 54 lesions (98%) completely responded (CR) to Rhenium-SCT® regardless of the dose received. Only a 91-year-old female patient presenting a 9.5-cm^2^ BCC (0.6-mm thickness) in the nose pyramid and left nose wing showed a small persistence of disease (patient classified as PR). This patient showed a suspicious area of persistent disease at dermoscopy, and the subsequent biopsy confirmed the presence of a small (1 cm^2^) basal cell carcinoma persistence located in the center of the field of irradiation. This lesion was surgically treated with a subsequent complete response and good cosmetic results. Twelve months after treatment, all the 41/41 evaluable lesions were free from relapse at dermoscopy. Twenty-four months after treatment 23/24 evaluable lesions were free of relapse while one patient treated for a 11.4-cm^2^ BCC (0.4-mm thickness) in the scalp showed a small (< 1 cm^2^) relapse in the edge of the field of irradiation. We scheduled this patient for a retreatment.

### Side effects

Different grades of early skin localized side effects started approximately after 14 days in all lesions and resolved completely within 90 days with excellent/good cosmetic results after 12–33 months. None of the patients reported significant pain or discomfort during or after the procedure. None of the patients showed any significant late side effect except dyschromia or slight atrophy of the skin or hair loss. None of the patients showed any significant late side effect during the follow-up period (3–33 months). Overall results, skin toxicity, cosmetic results, and follow-up are reported in Table 5.

**Table 5:** Overall results in the three groups of dose deescalation based on the target dose defined as the adsorbed dose to the deepest point of neoplastic invasion. Acute skin toxicity according to CTCAE 5.0 [10]. Cosmesis according to RTOG cosmetic scale [11]. Follow-up according to dermatologic examination and dermoscopy

Variables	Target dose 23 Gy (*n* = 27)	Target dose 35 Gy (*n* = 23)	Target dose 47 Gy (*n* = 10)	Total *n* 60 (100%)
Efifcacy Re SCT	21	23	10	54
CR	21	22	10	53 (98.2%)
PR	/	1	/	1 (1.8%)
Acute skin toxicity				60
G1	15	12	4	31 (51.6%)
G2	10	11	4	25 (41.6%)
G3	2	/	2	4 (6.6%)
Cosmesis (RTOG)				41
Good	4	3	4	11 (26.8%)
Excellent	5	19	6	30 (73.1%)
Follow-up				
12 months				41
CR	9	22	10	
Relapse	/	/	/	
24 months				24
CR	1	12	10	
Relapse		1		

In 56/60 lesions, early side effects, resolving within 32 days (mean 4 weeks), were consistent with skin erythema, faint or moderate edema, or little ulcerations (grades 1–2). In the remaining 4/60 lesions, these findings were more severe (grade 3) lasted up to 8–12 weeks (mean 10 weeks), but resolved within 90 days in all the cases. It is interesting to point out that two of these four lesions were located in the legs while the remaining two in the ear and face. Cosmetic results were evaluated in 41/60 evaluable lesions after a period of 12–33 months according to RTOG Cosmetic scale [12]. Thirty lesions were classified as excellent and 11 lesions as good.

The characteristics of these lesions are reported in Table 6**.**

**Table 6:** Comparison of G1–2 vs G3, early toxicity, lesions characteristic’s, and dose received

Early toxicity (CTCAE 5.0)	Duration early toxicity (weeks)	Cosmetic results (41 lesions)	Treated surface area (cm^2^)	Neoplastic thickness invasion (mm)	Volume (cm^3^)	Superficial dose * (Gy)	Mean dose ** (Gy)	Target dose *** (Gy)
56 lesions Grades 1–2	4 weeks	10 good 27 excellent	6.4	1.0	0.6	180	62	31
4 lesions Grade 3	10 weeks	3 good 1 excellent	15.8	1,6	2,9	250	76	33

Early toxicity measured according to CTCAE 5.0 [10]; Cosmetic results measured after 12–33 months according to Cosmetic scale RTOG [11]. *Superficial dose: adsorbed dose at 0.01 mm of neoplastic invasion. **Mean dose: adsorbed dose by the whole volume of the lesion. *** Target dose: adsorbed dose to the deepest point of neoplastic invasion. To note the significantly difference in treated surface area between the two groups

### Predictors of acute toxicity

Multivariate logistic regression analysis showed that both the mean dose and the treated surface areas were significantly and independently related to G3 acute toxicity. The AUC resulted 0.830 (*p* value = 0.0103) indicating that the mean dose and the treated surface areas are reliable predictors of toxicity. Univariate and multivariate logistic regression analysis are reported in Table 7.

**Table 7:** Univariate and multivariate logistic regression analysis of G1–G2 vs G3 toxicity according to CTCAE [10]

Univariate analysis	Variable	Coefficient	Standard error	*p* value
	Superficial dose * (Gy)	0.0053	0.0044	0.228
	Mean dose ** (Gy)	0.0232	0.0206	0.261
	Target dose to the deepest point of neoplastic invasion (Gy)	0.0172	0.0526	0.743
	Treated surface areas (cm2)	0.1187	0.0547	0.030
	Thickness neoplastic invasion (mm)	1.5165	0.9652	0.116
	Lesion volume (cm3)	0.8713	0.3834	0.023
Multivariate analysis §	Variable	Coefficient	Standard error	*p* value
	Treated surface areas (cm2)	0.2016	0.0759	0.0079
	Mean dose ** (Gy)	0.0545	0.0269	0.0426

^§^*p* = 0.0021

Variables were superficial maximal dose (Gy), mean dose (Gy), treated surface areas (cm^2^), thickness of neoplastic invasion (mm). In multivariate analysis only statistically significant variables are reported. *Superficial maximal dose: adsorbed dose at 0.01 mm of neoplastic invasion. **Mean dose: adsorbed dose by the whole volume of the lesion

## Discussion

The few papers published so far on the use of nonsealed brachytherapy with ^188^Re source in NMSC have shown very interesting results: Sedda et al. [7] treated 53 patients with NMSC with an acrylic ^188^Re matrix. In all cases, clinical remission occurred after 3 months while complete healing was obtained in 82% of the cases without any significant long-term side effect. The remaining 18% of patients required multiple applications. After a follow-up of 20–72 months, no clinical relapses were observed, and histology confirmed complete response in all cases. Authors did not report data about early or late toxicity. Carrozzo et al. [8] treated 15 patients with a histologically confirmed diagnosis of squamous cell cancer of the penis (SCCP). In this population, 12 healed, and two patients did not respond to ^188^Re brachytherapy. It is worth to underline that in these studies Authors delivered a standard dose of 50 Gy at the depth of 0.5 mm. This dosimetry was independent from the size and thickness of the lesions. Using this not personalized approach, the risk is to overtreat thin lesions and undertreat more thick lesions who may later deserve further treatments. Cipriani et al. [9] recently published a retrospective study on 52 patients showing 53 NMSC lesions and 2 extramammary Paget’s disease, treated with Rhenium-SCT®. In this study, authors delivered a standard dose of 50Gy at the deepest point of neoplastic invasion that ranged from 0.3 to 0.6 mm. Authors do not report data about early skin toxicity or cosmetic results, however long-term results showed a complete clinical remission in 36 lesions after 6 months and in 19 lesions after at least 12 months. This data confirm the already reported promising results published by the same authors [10].

Our preliminary findings confirm the promising results reported by the few works published so far**.** Histological specimens or dermoscopy, performed 6 months after treatment showed a complete remission in all 54 studied lesions except one in which, however, Rhenium-SCT® treatment reduced significantly the size of the lesion and made possible surgery with a subsequent complete response. Only one patient showed a small relapse in the edge of the field of irradiation in the scalp after 24 months.

In our study, we administered a standardized adsorbed dose to the deepest point of neoplastic invasion (target dose) and to the whole volume of the lesion (mean dose) in order to find optimal standard adsorbed dose able to treat the lesion in one single application, avoiding severe early, and late side effects. Given the not negligible incidence of early side-effects during our preliminary experience, we proceeded to a progressive reduction of the delivered dose after the treatment of the first 10 lesions where we observed a 20% G3 toxicity according to CTCAE 5.0. We established a dose deescalation protocol. The early toxicity reduced in the other two groups of dose deescalation (Tables 5 and 6).

Overall, in our population, the incidence of acute toxicity, classified as G3, is low but not negligible, 4/60 (6.6%). A possible explanation of such relatively high quote of G3 early toxicity may lie on the fact that we enrolled patients with very large lesions in terms of treated surface area and volume if compared with the lesions commonly treated with high dose brachytherapy [17–19]. Moreover, we administered the dose in a single fraction.

**Fig. 2:**
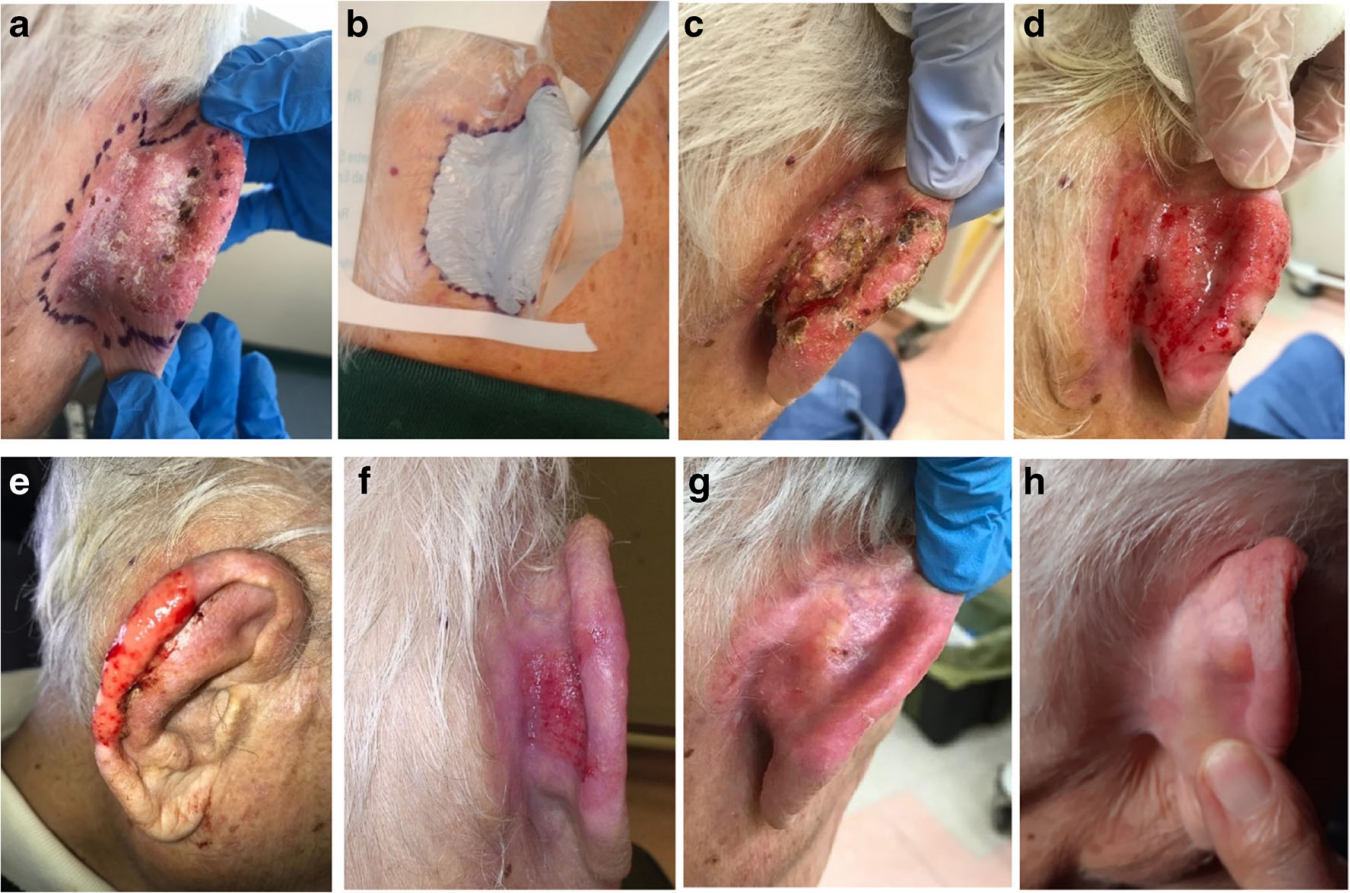
Male 93 years old with SCC of the right ear no previous therapies; area 36 cm^2^; thickness 2 mm according to multiple biopsies. **a** Day 0 before treatment, **b** application of ^188^Re resin (Rhenium-SCT® in whole surface of the lesion + 3-mm safe margins; administered dose 856 MBq; dose received from the surface 127 Gy; mean dose 35 Gy; dose received from the deepest point of lesion invasion (2 mm) 14 Gy; treatment time 130 min. **c** Day 14 toxicity grade 3 according to the CTCAE scale [11]. **d** and **e** The lesion after 30 days, **f** after 48 days, **g** after 90 days, **h** after 12 months. Dermoscopy was negative, and no biopsy was performed. The patient has been classified as complete responder. Excellent cosmetic results according to RTOG scoring criteria [12]

**Fig. 3:**
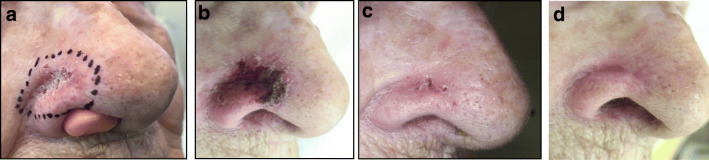
Female 92 years old with relapse of a BCC of the right wing of the nose; previously treated with Mohs surgery; area 3.3 cm^2^; thickness 0.4 mm according to multiple biopsies. **a** Day 0 before application of ^188^Re resin (Rhenium-SCT®; administered dose 330 MBq, dose received from the surface 96 Gy; mean dose 52 Gy; dose received from the deepest point of lesion invasion (0.4 mm) 36 Gy; treatment time 23 min, **b** day 14 toxicity grade 2 according to CTCAE scale [11] **c**) day 28 **d**) after six months dermoscopy and biopsy were negative. The patient was classified as complete responder. Excellent Cosmetic results according to RTOG scoring criteria [12]

**Fig. 4:**
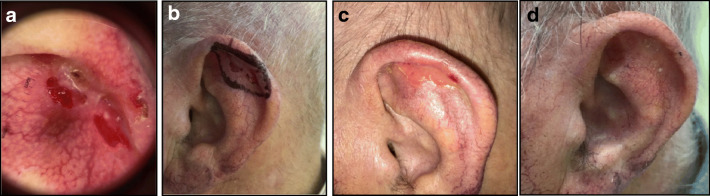
Male 87 years old with relapse of a ulcerated BCC of the left ear previously treated with cryotherapy; area 3.0 cm^2^; thickness 1.5 mm according to multiple biopsies. **a** Dermoscopy before the treatment, **b** day 0 before application of ^188^Re resin (Rhenium-SCT®); administered dose 213 MB; dose; received from the surface 265 Gy; mean dose 84 Gy; dose received from the deepest point of lesion invasion (1.5 mm) 38 Gy; treatment time 83 min, **c** day 14 early toxicity grade 2 according to the CTCAE scale [11], **d**) day 28 complete resolution of the wound. After six months dermoscopy and biopsy were negative. The patient was classified as complete responder. Excellent Cosmetic results according to RTOG scoring criteria [12]

**Fig. 5:**
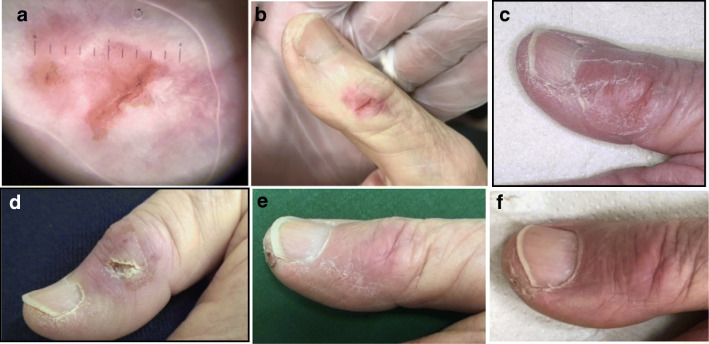
Male 84 years old relapse of SCC of the first finger of the right hand previously treated with cryotherapy and C02 laser; surface 2.5 cm^2^; thickness 0.6 mm according to multiple biopsies**. a** Dermoscopy before the treatment **b** day 0 before application of ^188^Re resin (Rhenium-SCT®); administered dose 300 MBq; dose received from the surface 125 Gy; mean dose 58 Gy; dose received from the deepest point of lesion invasion (0.6 mm) 37 Gy; treatment time 25 min, **c**) day 14 toxicity Grade 1 according to the CTCAE scale [11] **d**) day 28 **e**) day 60 **f**) day 90. After six months dermoscopy negative and biopsy was not performed. The patient was classified as complete responder. Excellent Cosmetic results according to RTOG scoring criteria [12]

However, it should be noted that all side effects were in most cases easily manageable, of short duration and not associated with pain, therefore without significant impact on patients’ quality of life (Figs. 2, 3, 4, and 5). Even in the four lesions showing a severe early and “long lasting” toxicity, we observed a complete healing of the wound within a maximum of 90 days. In one of these patients (Fig. 2), we observed a complete healing with excellent cosmetic results after 12 months. The reason of such phenomenon may lie in the fact that beta radiations deliver more than 90% of their energy in the first 2 mm of the skin in the epidermis, without deeper involvement of the derma making possible a fast recovering of the wound [6].

A rigorous statistical analysis of our data seems premature, and it is not in the aim of this preliminary publication. However at multivariate logistic regression analysis, the treated surface area and the mean dose received by the lesions are the variables associated with the presence of severe (G3) early side effects. We have also observed early G3 toxicity in 2/4 patients showing lesions in the legs. This confirms the findings of Ballester-Sánchez et al. [19] in BCC patients treated with electronic brachytherapy. In their population, authors found that one of the statistically significant predictors of toxicity was the location of the lesion in trunk or extremities. We cannot draw any conclusions based only on these few observed cases; however, this could be related to different thickness of the epidermis in the legs as opposed to the face or trunk. Another possible explanation may be the different vascularization of the skin, making it faster for a wound to recover in the face than in the extremities.

With regard to skin toxicity, G3 toxicity in our population was rare (6.6%), and it would premature to draw any reliable conclusions. No direct and linear correlation between the absorbed target doses and the onset of G3 skin toxicity was observed. The only two factors who presented a statistical significance in our analysis were the mean absorbed dose (which is closely correlated to the volume of lesions) and the treated surface area, while the target and the superficial absorbed doses did not show any significance at the uni- multivariate analysis. There could be other important factors, which may play a role in the onset of G3 toxicity: in example different radio-sensitivity and repair capacity between different patients, different epidermal thickness in different anatomical districts or different general health conditions. The current study reports a preliminary experience of a single center. More data is needed to better understand the optimal dose to administer able to achieve a complete response with one single application without significant early side effects. According to our preliminary observations, in our future trials, the personalized dose to deliver should mainly take into account the location, the surface area and mean absorbed dose and/or the volume of the lesions.

In conclusion, Rhenium-SCT® is a single-session painless technique, tailored on the patients and well-tolerated, that can probably provide better esthetic results compared to surgery and good efficacy. According to our preliminary experience, the main advantages of this technique are (1) the possibility to apply the treatment to lesions with complex geometry or where the surface is not planar (ears or wing nose for example) where other noninvasive techniques such as high dose rate brachytherapy or external beam RT may have some difficulties in delivering an homogeneous high dose rate to the whole lesion. (2) The suitability of this treatment for even large lesions (up to 36 cm^2^ in our population), where other treatment modalities may have some difficulties. (3) The possibility to use this technique in patients for whom surgical approach could be technically difficult or may result in a very poor outcome both from a functional or esthetic point of view. This is particularly true in patients in whom the lesions are located in the face, scalp, or ears.

A cost/benefit analysis of this treatment in comparison with other approaches is not in the aim of this preliminary study, however we would like to underline that Rhenium-SCT® can be carried out on an outpatient day service facility and involves a relatively limited number of staff before, during and after the treatment. The average duration of a single application is only 76 min and many patients (from three to six in our experience) can be treated at the same time.

### Main limitations

Despite this encouraging initial data, longer follow-up is needed in order to compare this treatment with its alternative competitors (brachytherapy or EBRT) by evaluating their long-term recurrence rate and, eventually, late side effects. A longer observation period is also needed to rule out the theoretical possible radio-induced local skin second malignancy.

A technical limitation is that the calculated absorbed doses (mainly mean and target) depend on the geometry of the tumors, which is frequently irregular. So, the exact volume of the lesions or the exact depth of neoplastic infiltration is difficult to calculate with high precision even according to multiple biopsies as we have performed in many cases. Accordingly, the data reported regarding the absorbed doses have to be taken with caution.

## Conclusions

High dose brachytherapy using a nonsealed ^188^Re resin (Rhenium-SCT®) is a noninvasive, easy to perform, and tolerable approach to treat NMSCs, and it seems to be an alternative when surgery or other Radiation therapy techniques are not possible, not recommended or refused by the patient. Our preliminary results are very encouraging, since in our population ^188^Re resin (Rhenium-SCT®) has shown to be effective in 98% of the treated patients. In the next future trials, larger populations and longer follow-up periods are needed to confirm these preliminary data and to find the optimal personalized dose in order to reduce early side effects.
